# Açai Berry Mitigates Vascular Dementia-Induced Neuropathological Alterations Modulating Nrf-2/Beclin1 Pathways

**DOI:** 10.3390/cells11162616

**Published:** 2022-08-22

**Authors:** Daniela Impellizzeri, Ramona D’Amico, Roberta Fusco, Tiziana Genovese, Alessio Filippo Peritore, Enrico Gugliandolo, Rosalia Crupi, Livia Interdonato, Davide Di Paola, Rosanna Di Paola, Salvatore Cuzzocrea, Rosalba Siracusa, Marika Cordaro

**Affiliations:** 1 Department of Chemical, Biological, Pharmaceutical and Environmental Sciences, University of Messina, Viale Ferdinando Stagno D’Alcontres 31, 98166 Messina, Italy; 2 Department of Veterinary Sciences, University of Messina, 98168 Messina, Italy; 3 Department of Pharmacological and Physiological Science, Saint Louis University School of Medicine, Saint Louis, MO 63104, USA; 4 Department of Biomedical, Dental and Morphological and Functional Imaging, University of Messina, Via Consolare Valeria, 98125 Messina, Italy

**Keywords:** vascular dementia, oxidative stress, autophagy, açai berry

## Abstract

The second-most common cause of dementia is vascular dementia (VaD). The majority of VaD patients experience cognitive impairment, which is brought on by oxidative stress and changes in autophagic function, which ultimately result in neuronal impairment and death. In this study, we examine a novel method for reversing VaD-induced changes brought on by açai berry supplementation in a VaD mouse model. The purpose of this study was to examine the impact of açai berries on the molecular mechanisms underlying VaD in a mouse model of the disease that was created by repeated ischemia–reperfusion (IR) of the whole bilateral carotid artery. Here, we found that açai berry was able to reduce VaD-induced behavioral alteration, as well as hippocampal death, in CA1 and CA3 regions. These effects are probably due to the modulation of nuclear factor erythroid 2-related factor 2 (Nrf-2) and Beclin-1, suggesting a possible crosstalk between these molecular pathways. In conclusion, the protective effects of açai berry could be a good supplementation in the future for the management of vascular dementia.

## 1. Introduction

In the Western world, vascular dementia (VaD) is the second-most common cause of organic acquired cognitive dysfunction; in several Asian nations, it is likely the main cause [1].

There are no viable pharmaceuticals approved for the treatment of VaD in any country despite the recent investments made in experimental and clinical neuroscience [2]. Smoking, not exercising, eating poorly, and being exposed to pollutants are all known to worsen the neuronal environment and increase oxidative stress, which can lead to the cognitive loss associated with aging and neurodegenerative disorders. Important brain areas like the hippocampus should be more vulnerable to oxidative and inflammatory stresses since these conditions might impair synaptic plasticity and memory by causing dendritic alteration and neuronal death. Furthermore, the brain is also subjected to an altered expression of two important neuronal markers of wellbeing: MAP-2 and β-Tubulin.

As it promotes microtubule stiffness, MAP2—which is found in cell bodies and dendrites—is regarded as a microtubule stabilizer. When expression is changed, MAP2 builds up into granules, causing neurotoxicity and neuronal degeneration. During cortical development, the tubulin family primarily expresses itself in post-mitotic neurons with a particular geographic and temporal expression pattern. Microtubules’ primary building blocks, tubulin, are essential for the mechanisms of the central nervous system’s (CNS) development, including neuronal migration and axonal guidance. The altered expression of these proteins may result in the death of neurons. The loss of MAP2 and β-Tubulin have been strongly associated with VaD [3,4]. 

Additionally, defective autophagy has been linked to increased oxidative stress in the brain, altering protein "quality control," accumulating undesired proteins and organelles in brain cells [5]. 

A fundamental leucine zipper redox-sensitive transcription factor called nuclear factor erythroid 2-related factor 2 (Nrf2) regulates the redox status of the cell under damaging stressors. Under normal circumstances, Kelch-like ECH-associated protein 1(Keap1), Nrf2’s inhibitor, anchors Nrf2 in the cytoplasm. However, during oxidative stress, Nrf2 separates from Keap1, moves into the nucleus, and interacts with the antioxidant response element (ARE) to control the production of antioxidant genes such heme oxygenase-1 (HO-1). Additionally, recent studies have shown that Nrf2 could control the initiation of autophagy, furthering its protective benefits [6,7,8]. In fact, inclusion bodies, which are pathogenic markers of many neurodegenerative disorders, are known to accumulate excessively as a result of dysfunctional autophagy. Numerous studies have demonstrated that entire fruits and dietary polyphenols such as anthocyanins, stilbenes, melatonin, kaemferol, quercetin, and resveratrol protect neurons by a modulation of autophagy process [9,10,11,12,13,14,15,16,17,18,19,20].

Scientists have recently developed an interest in açai seeds. The berry known as the açai berry has a variety of beneficial nutritional properties as well as some possible medicinal uses. This fruit has a tart flavor and is produced by the *Euterpe oleracea* palm, which can only be found in the Amazon. For millennia, Amazonian Indians have employed the açai fruit, which is regarded as a high-energy meal, as a food source and a natural remedy for a number of illnesses [21,22,23,24,25,26,27,28,29]. 

The pulp of the açai fruit has been extensively researched due to its high bioactive nutritional and phytochemical content. Açai berry pulp composition contains a wide range of physiologically active phytochemicals as well as significant amounts of mono- and polyunsaturated fatty acids, which are uncommon in most fruits and other berries. Phytochemicals such proanthocyanidins, anthocyanins, and other flavonoids are present in açai pulp. Additionally, phytochemical analyses revealed that the açai berry contains a significant amount of luteolin, quercetin, dihydrokaempferol, and chrysoerial, among other polyphenolics, as well as a variety of anthocyanins, including cyanidin, delphinidin, malvidin, pelargonidin, and peonidin. Açai fruit pulp included five different types of carotenoids, including carotene, lycopene, astaxanthin, lutein, and zeaxanthin [30].

Numerous pharmacological benefits of açai berry extract and its bioactive components include anti-inflammatory and anti-anxiety properties through modulation of oxidative stress, inflammation, autophagy, and Nrf2 expression in the hippocampus and frontal cortex [31,32,33,34,35,36,37,38,39]. However, more data are needed to confirm the neuroprotective, anti-inflammatory and antioxidant effect. For this reason, we used a consolidated murine model of VaD to investigate the potential beneficial effects of açai supplementation and the molecular way by which it acts.

## 2. Materials and Methods

### 2.1. Animals 

CD1 male mice (8-week-old, 18–24 g) were acquired from Envigo (Milan, Italy) and located in a controlled environment. All animal experiments complied with the new Italian regulations (D. Lgs 2014/26), EU regulations (EU Directive 2010/63) and the ARRIVE guidelines.

### 2.2. Experimental Design and Groups

Mice were subjected to temporary bilateral carotid occlusion as previously described by Cordaro et al. [40]. Briefly, following anesthesia, the bilateral carotid arteries underwent three cycles of ligation and release lasting a total of 10 min each. After the threading was taken out, the incision was stitched up. After 15 days of induction, the mice were sacrificed, and their brains were removed and processed for histological and biochemical analysis [40,41].

Mice were arbitrarily divided into groups:

Group 1: Sham = mice were subjected to the surgery without carotid arteries ligation and treated daily orally with saline (vehicle) for 15 days.

Group 2: Sham + açai berry = same as above, but açai berry at a dose of 500 mg/kg, dissolved in saline, was administered orally once a day for 15 days (data not shown).

Group 3: VaD = mice were subjected to the VaD surgery described above and treated with vehicle.

Group 4: VaD + açai berry = same as above, but açai berry at a dose of 500 mg/kg was administered daily orally for 15 days.

A total of six samples was used for each technique. Freeze-dried açai extract was dissolved in distilled water. This substance (Cas Number: 879496-95, 906351-38-0) was purchased from Farmalabor Srl (Canosa di Puglia, Barletta, Italy) (see Supplementary File for technical datasheet). The dose and the route of administration of açai berry were chosen based on our previous study [42]. No significant difference was found between the sham and sham + açai berry, so only data regarding the sham groups are shown.

### 2.3. Behavioral Tests

#### 2.3.1. Open Field Test (OFT)

Locomotor activity and anxiety-like behavior were monitored by the OFT. Each mouse was carefully placed in the center of the box after training, and any action was counted as a line crossing if a mouse moved all four paws out of one square and into another [43,44]. 

#### 2.3.2. Novel Object Recognition (NOR) Test 

The spontaneous inclination of mice to spend time investigating a novel object or a familiar one was examined with the NOR test. After a training session, mice were switched out in the box for a five-minute exam, during which the examiner, at random, switched out one of the familiar objects for a new one. The amount of time the mouse spent investigating each item was measured [41,45]. 

#### 2.3.3. Social Interaction Test (SIT)

A three-chambered device was used to create the social interaction test as previously described. The number of active interactions and the duration of contacts were measured [41]. 

### 2.4. Histopathological Evaluation 

Brains were stained with hematoxylin/eosin (H/E) or cresyl-violet and blindly analyzed using light microscopy LeicaDM6 connected to an imaging system (LasX Navigator)(Leica Microsystem, Buccinasco, Italy) by two investigators without knowledge of the experimental groups [46,47,48]. The severity of brain injury and numbers of dead cells was assessed as previously described [40,41].

### 2.5. Immunohistochemical Evaluation 

Brain sections were incubated with anti-NRF2 antibody (1:250, Santa Cruz Biotechnology) as previously described by Cordaro et al. [49,50]. At the end of the protocol, the digital photos were analyzed by two observers blinded to the treatment as previously made in our laboratory [51,52,53,54,55]. 

### 2.6. Terminal Deoxynucleotidyl Nick-End Labeling (TUNEL) Assay

TUNEL staining for apoptotic cell nuclei and DAPI staining for all cell nuclei were performed in brain sections as described previously [56,57,58,59]. 

### 2.7. Immunofluorescence Evaluation

Brain sections were incubated with the following primary antibodies: HO-1 (Santa Cruz Biotechnology; 1:50 in PBS, *v*/*v*) or β-Tubulin (Santa Cruz Biotechnology, Heidelberg, Germany; 1:50) or MAP-2 (Santa Cruz Biotechnology; 1:50), as previously described [60,61,62,63,64,65]. After the incubation, sections were washed with PBS and incubated with secondary antibody FITC-conjugated anti-mouse Alexa Fluor-488 (1:2000 *v*/*v* Molecular Probes, UK) and 40,60-diamidino-2-phenylindole (DAPI; Hoechst, Frankfurt; Germany). Sections were observed and photographed using a Leica DM6 microscope (Leica Microsystems SpA, Milan, Italy) [66,67,68,69,70]. 

### 2.8. RT-qPCR

Total RNA was isolated from the hippocampus using TRIzol Reagent (Invitrogen, USA) following the manufacturer’s recommendations. Reverse transcription of cDNA was performed according to the manufacturer’s instructions as previously described [71]. The following primer sequences (5′–3′) were used: Beclin-1 (F) GCTGTAGCCAGCCTCTGAAA (R) AATGGCTCCTGTGAGTTCCTG; LC3B (F) GGGACCCTAACCCCATAGGA (R) TCTCCCCCTTGTATCGCTCT; P62 (F) ACTGCTCAGGAGGAGACGAT (R) CCGGGGATCAGCCTCTGTAG; Bcl-2 (F) GCGTCAACAGGGAGATGTCA (R) GCATGCTGGGGCCATATAGT; Bax (F) CTGGATCCAAGACCAGGGTG (R) GTGAGGACTCCAGCCACAAA β-Actin (F) ACACTCTCCCAGAAGGAGGG (R) TTTATAGGACGCCACAGCGG. Relative expression of mRNA was calculated by the delta–delta C_T_ method [72,73]. All values for the mRNA species were normalized to β-actin.

### 2.9. Western Blot Analysis of Cytosolic and Nuclear Extracts

Extracts from cytosol and nucleus were prepared as previously described and incubated with the antibodies anti-NRF-2 (1–500, SCB, Heidelberg, Germany, #sc-365949), anti-heme oxygenase 1 (HO-1; 1-500, SCB, Heidelberg, Germany, #sc-136960), anti-β-tubulin (1–500, SCB, Heidelberg, Germany, #sc-166729), and anti-MAP2 (1–500, SCB, Heidelberg, Germany, #sc-74421) in 1× PBS, 5% *w*/*v* non-fat dried milk and 0.1% Tween-20 at 4 °C overnight [46,47,48,74,75,76,77,78,79,80,81,82,83]. For the cytosolic and nuclear fraction, blots were also probed with β-actin and lamin A/C protein to ensure that they were filled with equivalent amounts of proteins (1:500; Santa Cruz Biotechnology). Signals were detected as previously described in our works [49,84,85,86]. 

### 2.10. Materials

All compounds were purchased from Sigma-Aldrich (Milan, Italy). All solutions used for in vivo infusions were prepared using nonpyrogenic saline (0.9% NaCl; Baxter Healthcare Ltd., Thetford, Norfolk, UK). 

### 2.11. Statistical Evaluation

In this study, the data are expressed as the average ± SEM and represent at least three experiments carried out on different days. For in vivo studies, N represents the number of animals used. The number of animals used for in vivo studies was determined by G*Power 3.1 software (Die Heinrich-Heine-Universität Düsseldorf, Düsseldorf, Germany). The images used in the histology, immunofluorescence, and immunohistochemistry came from at least three independent investigations. For multiple comparisons, a one-way ANOVA was employed, then a Bonferroni post hoc analysis; 0.05 was a significant *p*-value.

## 3. Results

### 3.1. Açai Berry Improve Behavioral Changes Vascular Dementia-Induced

**Figure 1:**
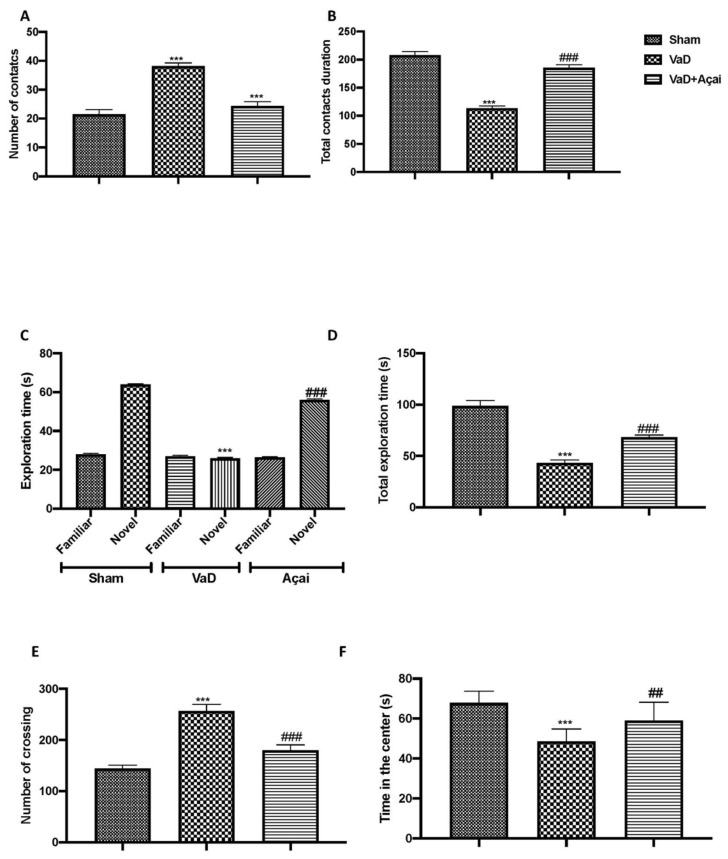
Açai berry improves vascular-dementia-induced behavioral changes. (**A**) Number of contacts and (**B**) total contact duration measured during social interaction test; (**C**) time spent to explore familiar or novel object, and (**D**) total exploration time measured during novel object recognition test; (**E**) number of crossing and (**F**) time in the center measured during open field test. Values are means ± SEM of six mice for all groups. See manuscript for further details. *** *p* < 0.001 vs. sham; ### *p* < 0.001 vs. VaD; ## *p* < 0.01 vs. VaD.

We observed that after the unfamiliar mouse was placed in the three-chamber test arena, the number of contacts (Figure 1A) considerably increased while the total duration of contacts (Figure 1B) decreased compared to the control group. On the other hand, after oral açai administration for 15 days at the dose of 500 mg/kg, we noticed that the behavior was more similar to the control group than the vehicle group. Additionally, we assessed alterations in cognitive function using the novel object recognition test (Figure 1C,D). Controls and VaD animals did not significantly differ in their exploration of items during training; however, 15 days after carotid artery ligation, VaD animals dramatically decreased their preference for the novel object, indicating compromised cognitive function. However, after taking açai orally for 15 days at a dose of 500 mg/kg, we found that the behavior was more like the control group than the vehicle group.

### 3.2. Açai Berry Limits Histological Changes Vascular Dementia-Induced

**Figure 2:**
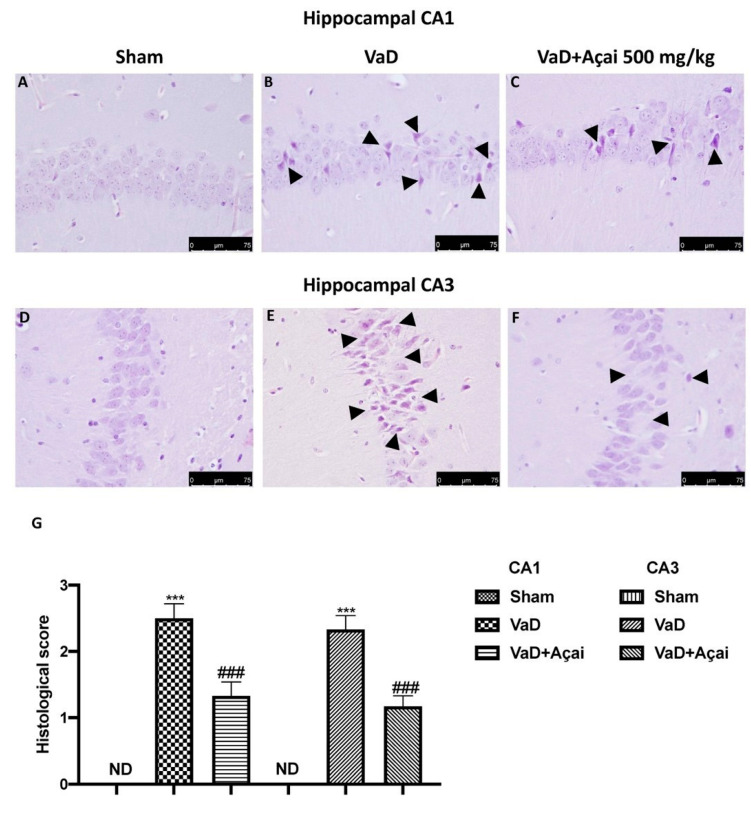
Açai berry limits vascular-dementia-induced histological changes. Hippocampal CA1 figure of Sham (**A**), VaD (**B**), Açai Berry (**C**); Hippocampal CA3 figure of Sham (**D**), VaD (**E**), Açai Berry (**F**); Histological score (**G**). Arrows indicate altered neurons and loss of hippocampal architecture. Figures are representative from least three experiments. Values are means ± SEM of six mice for all groups. See manuscript for further details. *** *p* < 0.001 vs. sham; ### *p* < 0.001 vs. VaD.

At 15 days after the induction of vascular dementia, all slices were stained with H&E to observe the severity of the hippocampal areas’ damage. As showed in Figure 2B (for CA1 region) and 2E (for CA3 region), the hippocampal regions of the animals that had suffered VaD damage exhibited disorderly and rigidly labeled neurons. After daily oral administration of açai, brain sections showed a clear reorganization of the hippocampal CA1 and CA3 regions with an increased number of hippocampal neurons (Figure 2C for CA1 region, 2F for CA3 region). No alteration was observed in sham animals (Figure 2A for CA1 region, 2D for CA3 region; see histological score G).

### 3.3. Açai Berry Limits Neuronal Death in the Hippocampus

**Figure 3:**
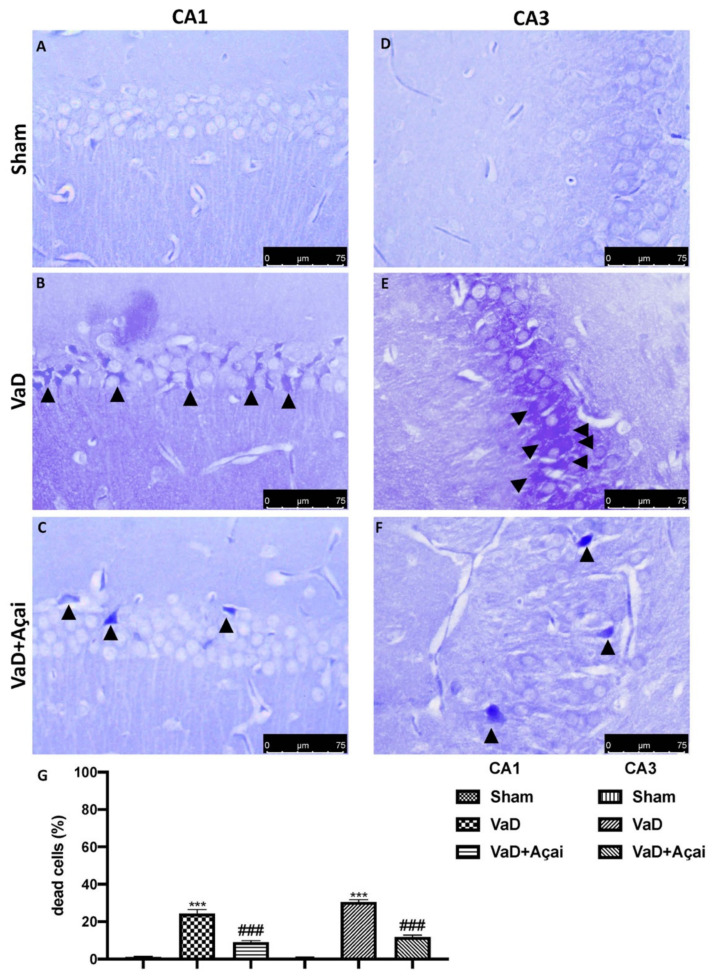
Açai berry limits neuronal hippocampal deaths. Hippocampal CA1 figure of Sham (**A**), VaD (**B**), Açai Berry (**C**); Hippocampal CA3 figure of Sham (**D**), VaD (**E**), Açai Berry (**F**); count of dead cells (**G**). Arrows indicate death neurons in both hippocampal sections. Figures are representative from at least three experiments. Values are means ± SEM of six mice for all groups. See manuscript for further details. *** *p* < 0.001 vs. sham; ### *p* < 0.001 vs. VaD.

**Figure 4:**
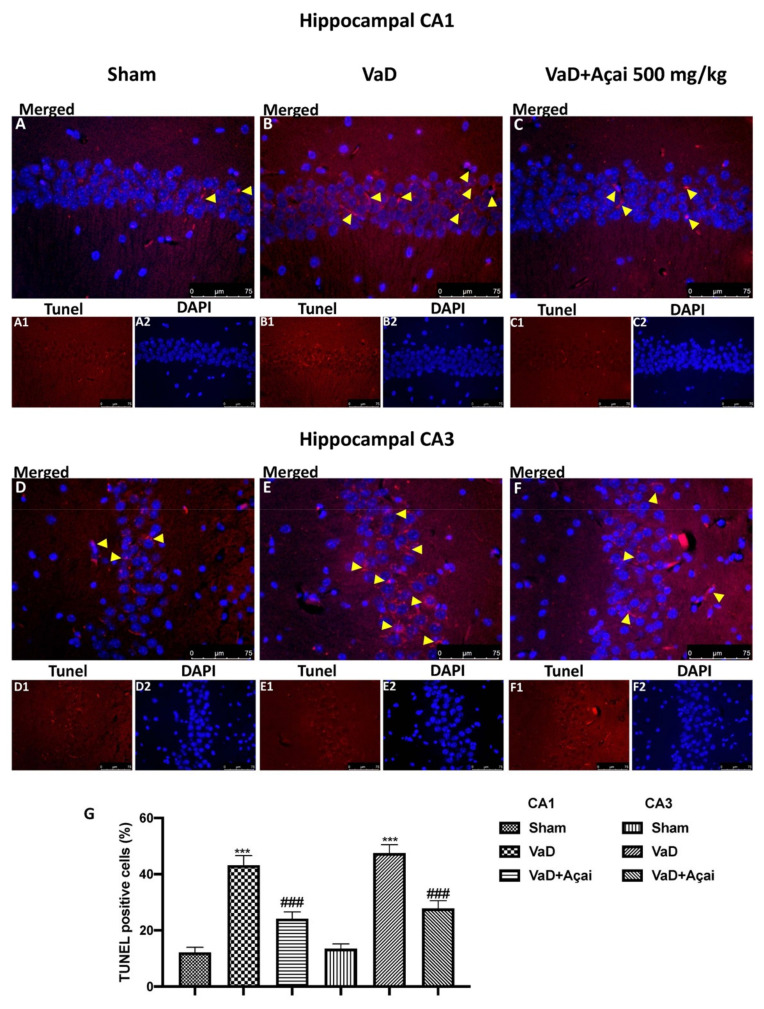
Açai berry reduces hippocampal TUNEL positive staining. Hippocampal CA1 figure of Sham (**A**,**A1**,**A2**), VaD (**B**,**B1**,**B2**), Açai Berry (**C**,**C1**,**C2**); Hippocampal CA3 figure of Sham (**D**,**D1**,**D2**), VaD (**E**,**E1**,**E2**), Açai Berry (**F**,**F1**,**F2**); count of dead cells (**G**). Arrows indicate TUNEL positive cells, merged with DAPI. Figures are representative from at least three experiments. Values are means ± SEM of six mice for all groups. See manuscript for further details. *** *p* < 0.001 vs. sham; ### *p* < 0.001 vs. VaD.

Next, we investigated whether açai berry could attenuate neuronal death in the hippocampal structure, which is an essential feature in VaD patients [87]. For this, we used cresil violet and TUNEL staining to assess the effects of açai berry administration on the loss of pyramidal neurons in the hippocampus, especially in the CA1 and CA3 subregion. As showed in Figure 3B,E and in Figure 4B,E, the vehicle group was characterized by an intense neuronal loss due to the death of pyramidal neurons in both CA1 and CA3 regions compared to the control group, Figure 3A,D and in Figure 4A,D. On the other hand, daily administration of açai berry at the dose of 500 mg/kg was able to significantly reduce neuronal death in both regions (Figure 3C,F and Figure 4C,F; see relative score, Figure 3G and Figure 4G).

### 3.4. Açai Berry Modulates Nrf-2 Pathways

**Figure 5:**
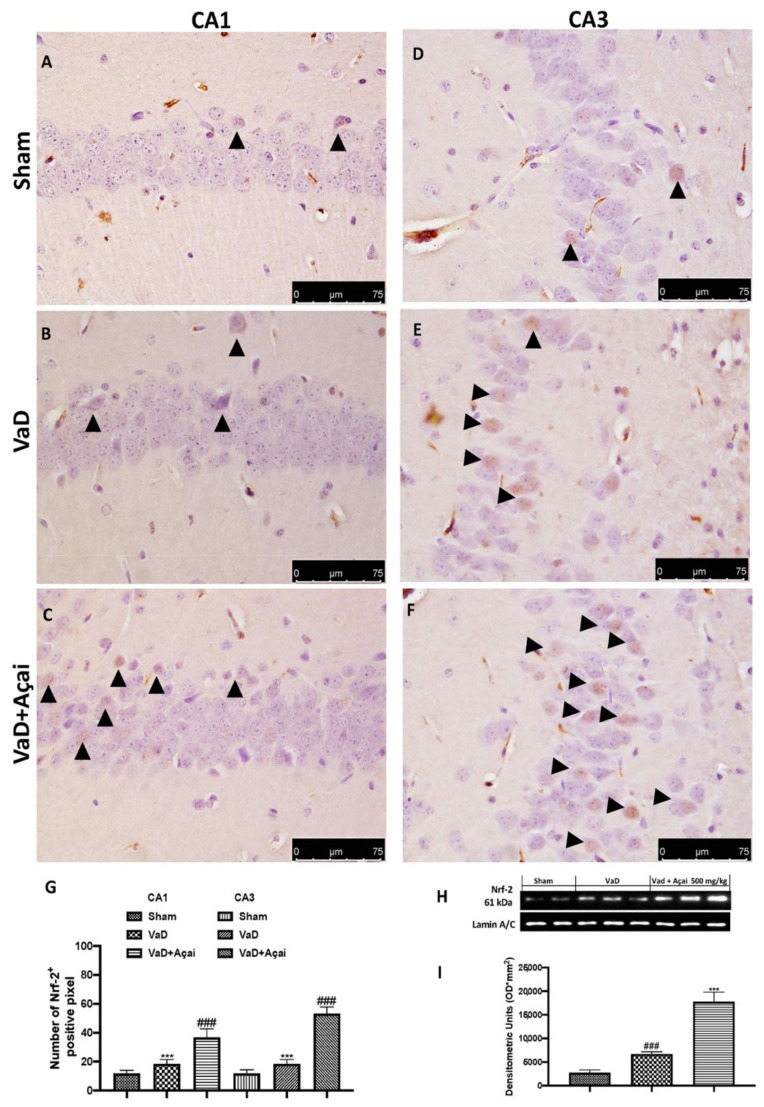
Açai berry improve NRF-2 expression. Hippocampal CA1 figure of Sham (**A**), VaD (**B**), Açai Berry (**C**); Hippocampal CA3 figure of Sham (**D**), VaD (**E**), Açai Berry (**F**); Number of Nrf-2 positive pixel (**G**); (**H**) western blot of NRF-2 expression and (**I**) relative densitometric analysis. Arrows indicate Nrf-2 positive cells. Figures are representative from at least three experiments. Values are means ± SEM of six mice for all groups. See manuscript for further details. *** *p* < 0.001 vs. sham; ### *p* < 0.001 vs. VaD.

**Figure 6:**
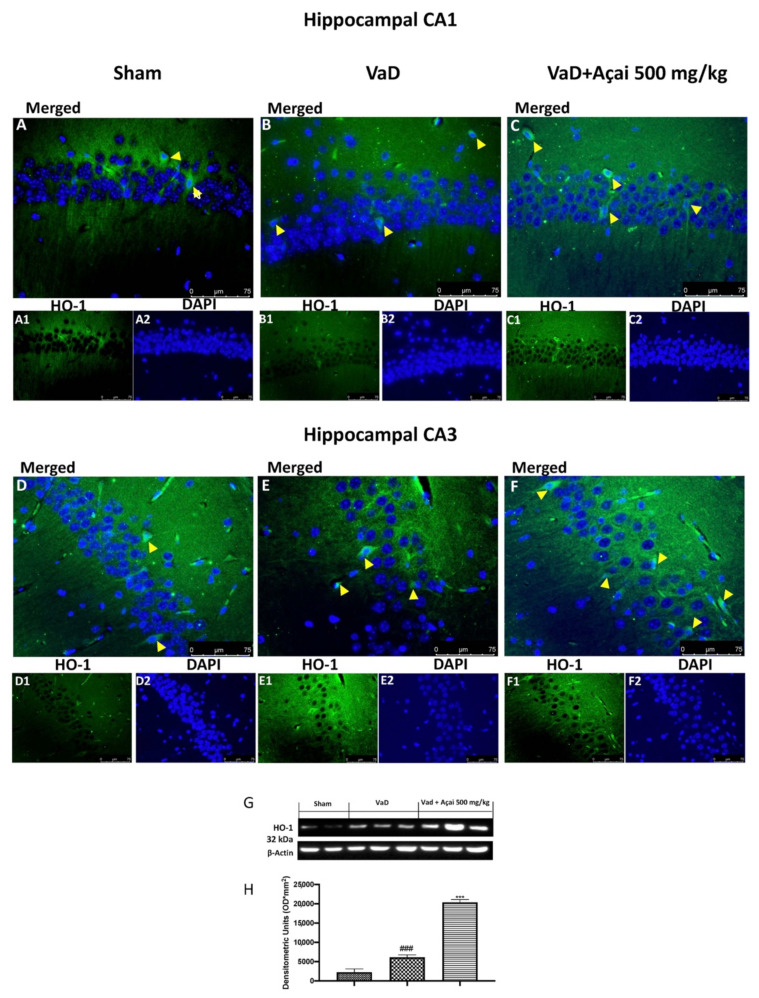
Açai berry increase HO-1 expression. Hippocampal CA1 figure of Sham (**A**,**A1**,**A2**), VaD (**B**,**B1**,**B2**), Açai Berry (**C**,**C1**,**C2**); Hippocampal CA3 figure of Sham (**D**,**D1**,**D2**), VaD (**E**,**E1**,**E2**), Açai Berry (**F**,**F1**,**F2**); (**G**) western blot of HO-1 and (**H**) relative densitometric analysis. Arrows indicate HO-1-DAPI merged cells in both hippocampal sections. Values are means ± SEM of six mice for all groups. Figures are representative from at least three experiments. See manuscript for further details. *** *p* < 0.001 vs. sham; ### *p* < 0.001 vs. VaD.

In our work, by immunohistochemistry staining and western blot analysis, we found an increase in NRF-2 expression after VaD induction (see Figure 5B for CA1, Figure 5E for CA3, Figure 5G for quantification, and Figure 5H,I for western blot) compared to the control group (see Figure 5A for CA1, Figure 5D for CA3, Figure 5G for quantification, and Figure 5H,I for western blot). As expected, after açai berry administration, the physiological response was significant (see Figure 5C for CA1, Figure 5F for CA3, Figure 5G for quantification, and Figure 5H,I for western blot). The same trend was also observed in the expression of HO-1, the most important enzyme regulated by Nrf-2 (see Figure 6A,A1,A2 for CA1 region and Figure 6D,D1,D2 for CA3 region of Sham group, Figure 6B,B1,B2 for CA1 region and Figure 6E,E1,E2 for CA3 region of VaD group, Figure 6C,C1,C2 for CA1 region and Figure 6F,F1,F2 for CA3 region of Açai Berry group, and Figure 6H,I for western blot).

### 3.5. Açai Berry Modulates Apoptotic and Autophagic Pathways

**Figure 7:**
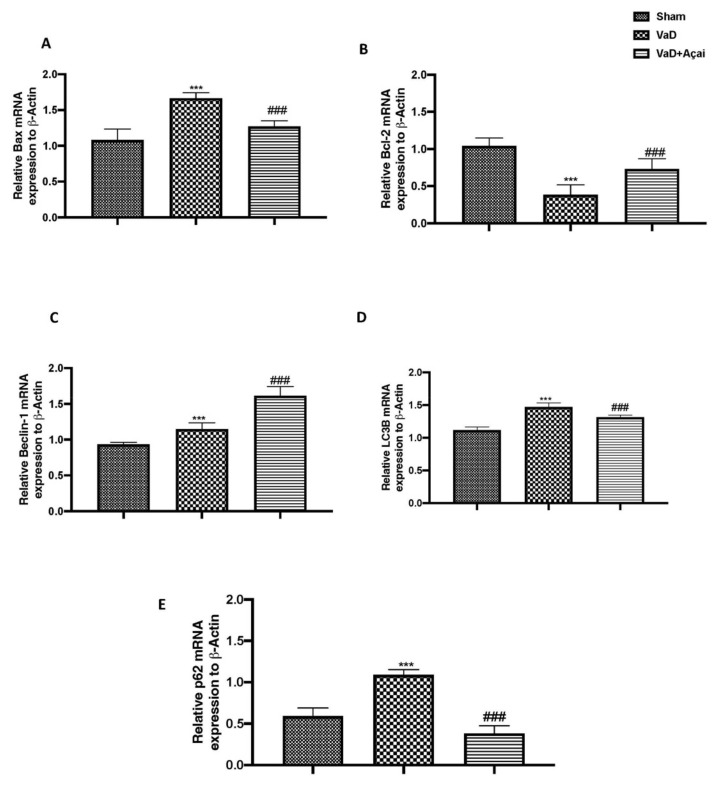
Açai berry modulates apoptotic and autophagic pathways. (**A**) Bax, (**B**) Bcl-2, (**C**) Beclin-1, (**D**) LC3B, (**E**) p62, mRNA levels in the hippocampus. Figures are representative from at least three experiments. Values are means ± SEM of six mice for all groups. See manuscript for further details. *** *p* < 0.001 vs. sham; ### *p* < 0.001 vs. VaD.

To further investigated neuronal death, we made RT-qPCR for Bax, and Bcl-2 and we found that after VaD induction there was a significant increase in Bax expression and a significant decrease in Bcl-2 expression compared to sham group (Figure 7A,B). Vice versa, the daily oral administration of açai berry at a dose of 500 mg/kg was able to restore both apoptotic markers to almost physiological values (Figure 7A,B). Autophagy and apoptosis interact in a complex way because, depending on the biological context, it can either promote cell survival or cell death [88]. In our study, açai berry supplementation increased autophagy in the hippocampus by activating Beclin1 (Figure 7C) decreasing LC3B turnover (Figure 7D), which led also to a decrease in p62 accumulation (Figure 7E).

### 3.6. Açai Berry Mitigates Brain Structure Change VaD-Induced

**Figure 8:**
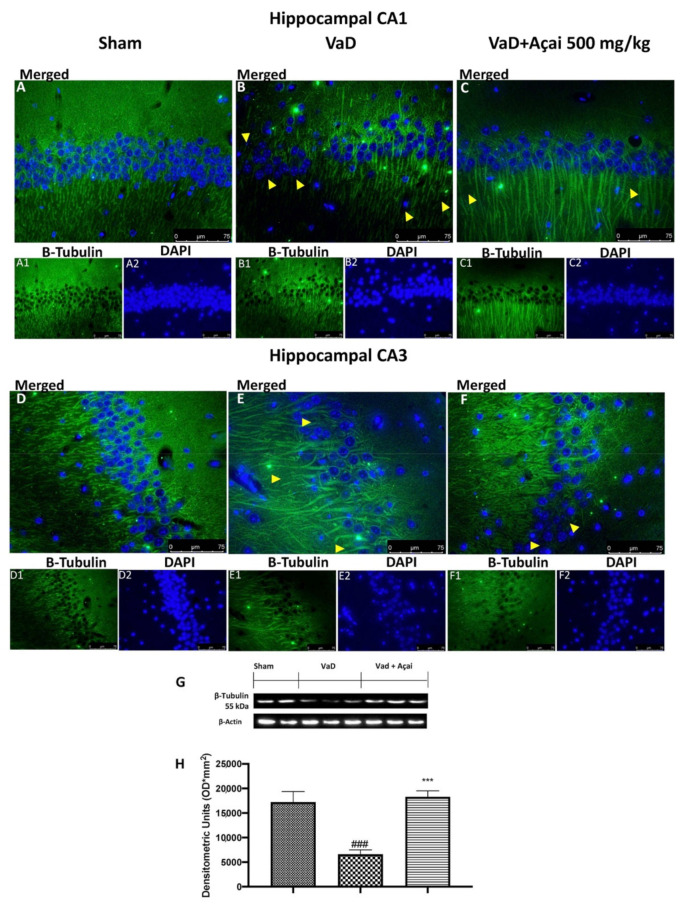
Açai berry mitigates β-tubulin alteration. Hippocampal CA1 figure of Sham (**A**,**A1**,**A2**), VaD (**B**,**B1**,**B2**), Açai Berry (**C**,**C1**,**C2**); Hippocampal CA3 figure of Sham (**D**,**D1**,**D2**), VaD (**E**,**E1**,**E2**), Açai Berry (**F**,**F1**,**F2**); β-tubulin western blot (**G**) and relative densitometric analysis (**H**). Arrows indicate the alteration or absence of β-tubulin. Values are means ± SEM of six mice for all groups. Figures are representative from at least three experiments. See manuscript for further details. *** *p* < 0.001 vs. sham; ### *p* < 0.001 vs. VaD.

**Figure 9:**
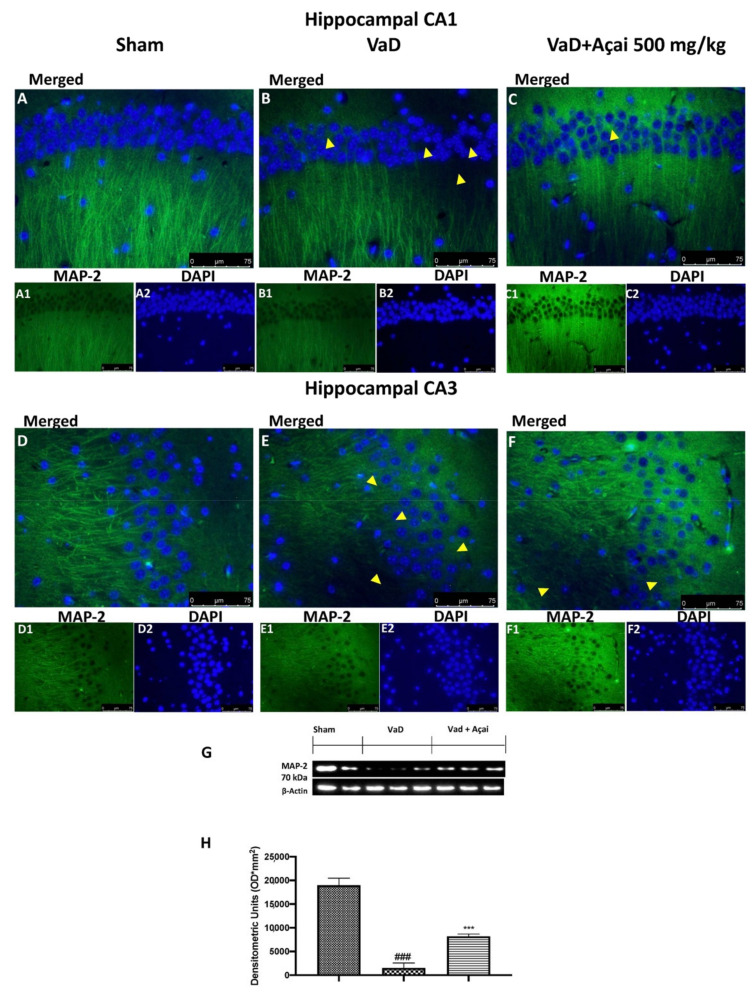
Açai berry mitigates MAP-2 alteration. Hippocampal CA1 figure of Sham (**A**,**A1**,**A2**), VaD (**B**,**B1**,**B2**), Açai Berry (**C**,**C1**,**C2**); Hippocampal CA3 figure of Sham (**D**,**D1**,**D2**), VaD (**E**,**E1**,**E2**), Açai Berry (**F**,**F1**,**F2**); MAP-2 western blot (**G**), and relative densitometric analysis (**H**) Arrows indicate the alteration or absence of MAP-2. Values are means ± SEM of 6 mice for all groups. Figures are representative from at least three experiments. See manuscript for further details. *** *p* < 0.001 vs. sham; ### *p* < 0.001 vs. VaD.

It is well-known that reduced levels of tubulin were found in patients with VaD [3]. In our study, we detected by immunofluorescence a significant decrease of b-tubulin in animals subjected to the operation compared to Sham group in both hippocampal regions. This decrease was significantly reduced after daily administration of açai berry at the dose of 500 mg/kg (see Figure 8A,A1,A2 for CA1 region and Figure 8D,D1,D2 for CA3 region of Sham group, Figure 8B,B1,B2 for CA1 region and Figure 8E,E1,E2 for CA3 region of VaD group, Figure 8C,C1,C2 for CA1 region and Figure 8F,F1,F2 for CA3 region of Açai Berry group). The same result was also observed with the immunofluorescence analysis of MAP-2. In this case as well, we observed a significant reduction of MAP-2 expression after VaD induction compared to the control group, which was significant restored after açai berry administration (see Figure 9A,A1,A2 for CA1 region and Figure 9D,D1,D2 for CA3 region of sham group, Figure 9B,B1,B2 for CA1 region and Figure 9E,E1,E2 for CA3 region of VaD group, Figure 9C,C1,C2 for CA1 region and Figure 9F,F1,F2 for CA3 region of Açai Berry group). These results were also confirmed by western blots analysis of β-tubulin and MAP-2 (see Figure 8G,H and Figure 9G,H, respectively).

## 4. Discussion

The pathologic disease known as VaD, which affects the elderly, is characterized by disturbed cerebral blood flow. Loss of judgment, reason, and especially cognitive and memory abilities are its symptoms [89]. Advanced age, previous stroke episodes, hypertension, higher blood pressure, diabetes mellitus, smoking, high hematocrit, changes in hemostasis, dyslipoproteinemia, high alcohol consumption, aspirin use, psychological stress in early life, occupational exposure to pesticides, herbicides, liquid plastic or rubber, and premorbid personality traits made the subjects more susceptible to developing VaD [90]. Neuronal degeneration, axonal injury, and white matter injury in VaD are caused by a number of processes. They frequently share vascular dysfunction-induced common molecular and regulatory pathways of neuronal survival and death. Of these, oxidative stress, apoptosis, and autophagy pathways typically demonstrate different processes of their own. Along with age and other risk factors acting in concert, a crosstalk between these signaling pathways contributes to myelinated axonal damage and cerebrovascular diseases. The diseased characteristics cause changes in the health of the neuronal population as well as a series of harmful events and white matter destruction. Cognitive impairment and brain atrophy are the results of these diseased traits. 

Although numerous papers describe the pathologic mechanisms behind vascular dementia, it is uncertain how the signaling pathways involved in vascular neuropathologies and cerebrovascular dysfunction interact with one another [91]. Accumulated data from numerous studies show that supplementing the diet with fruits, nuts, and vegetables of vibrant colors enhances the motor, memory, and cognitive abilities of both people and animals [5,66,67,68,69,70]. The antioxidant defense system may be compromised by increased oxidative stress brought on by a poor diet. On the other hand, a nutritious diet that is well-balanced and rich in a variety of nutrients, such as numerous servings of fruits and vegetables, low dosages of omega-3 fatty acids, tea, coffee, and wine, may provide neuroprotection [92,93]. 

The new food that is discussed in this paper is a fruit from the Euterpe genus of tropical palm trees that is native to South America and is commonly referred to as “Açai.” Researchers have been investigating *Euterpe oleracea* due to its high antioxidant content when compared to other fruits and berries. The composition of açai pulp has also been studied, and it contains a number of phytochemicals that have physiological activity. Numerous studies have demonstrated the neuroprotective properties of açai berries [30]. The causes of many of these diseases are multifactorial, including oxidative stress, chronic neuroinflammation, excitotoxicity, mitochondrial dysfunction, alteration in autophagy, abnormal protein accumulation in brain tissues, and other cellular aetiologias. These diseases are brought on by a combination of aging, genetic disorders, and exposure to one or more environmental factors. Açai berry extracts have been shown in studies to have neuroprotective effects by, among other things, restoring calcium homeostasis and mitochondrial function, preventing the formation of toxic protein aggregates, and demonstrating antioxidant and anti-inflammatory activities. Additionally, açai fruit has anticonvulsant and antidepressant effects that may be beneficial for those with certain neurological conditions [94,95,96,97,98,99,100,101]. 

Keeping this aim in our mind, we decided to explore the neuroprotective effects of açai berry administration in a murine model of VaD. Depressive, anxious, or apathetic mood swings are common in people with vascular dementia. A person with vascular dementia may start acting strangely as their condition worsens. For instance, they might become angrier, hostile, or perplexed [102,103]. In our study, using different behavioral tests, we found that açai supplementation was able to reduce VaD-induced behavioral decline. It is well-known that a strong relationship exists between cognitive impairment and neuronal loss in the hippocampus. For this, we investigated by different staining what happen in CA1 and CA3. In particular, we found that both hippocampal sections showed, after VaD induction, a significant increase in altered and dead neurons, compared to Sham animals. After açai berry supplementation at the dose of 500 mg/kg, we notice an improvement in histological alteration as well as a decrease in dead neurons in both hippocampal regions.

Nrf2 is the master regulator of redox status and controls the transcription of a panel of antioxidative and anti-inflammatory genes. By cooperating with NF-κB (nuclear factor κB), Nrf2 also coordinates cellular oxidative and inflammatory balance. Nrf2 deficiency is linked to aging, and mounting evidence supports Nrf2’s role in slowing the VaD process. By reducing neuroinflammation and oxidative stress, targeting Nrf2 has become a popular strategy for treating neurodegenerative diseases [104,105,106]. In our study, we found that açai berry supplementation was able to improve physiological anti-oxidant defense, improving Nrf-2 expression as well as HO-1, reducing oxidative stress. Additionally, recent studies suggest that autophagy and the Kelch-like ECH-associated protein 1 (Keap1-Nrf2) signaling pathway cooperate to avoid oxidative injury in the brain, and that p62 phosphorylation activates Nrf2 in response to oxidative stress. This is particularly intriguing because recent articles have demonstrated dynamic Beclin-1 alterations in the hippocampus of male mice with VaD. Beclin-1 is a crucial regulator of several trafficking pathways, including autophagy and receptor recycling in microglia. [71,107]. Additionally, it has also been demonstrated that Beclin 1 regulates signaling through the neuroprotective TGF-β pathway by decreasing TGF-β1 receptor II (TBRII). TGF-β1 plays a key function in hippocampus synaptic plasticity, memory, and neuronal survival. It also regulates brain homeostasis [108,109,110]. In our study, we found that açai berry restored autophagic flux at physiological levels. Interesting studies have shown that the altered expression of MAP-2 and β-tubulin leads to neuronal death; such results could be used in the future target for neuroprotective therapy targeting [111,112,113,114]. In our work, we found that açai berry stops the process of cell death and neurodegeneration and restores the microtubule network.

## 5. Conclusions

In conclusion, we demonstrate for the first time that açai berry supplementation at a dose of 500 mg/kg was able to enhance the physiological anti-oxidant defense, suggesting a possible protective role during VaD events.

## Data Availability

The data used to support the findings of this study are available from the corresponding author upon request.

## Supplementary Materials

The following are available online at https://www.mdpi.com/article/10.3390/cells11162616/s1, Supplementary File: Technical Datasheet of EUTERPE OLERACEA P.E. 10% POLYPHENOLS.
