# Supplementary material for: Açai Berry Mitigates Vascular Dementia-Induced Neuropathological Alterations Modulating Nrf-2/Beclin1 Pathways
Source: Cells. 2022 Aug 22;11(16):2616. doi: 10.3390/cells11162616 (PMC9406985; doi:10.3390/cells11162616)
Supplement: Supplementary file 1 [file cells-11-02616-s001.zip › cells-1863455-supplementary.PDF]

FONTANA
standardised natural active principles

SCHEDA TECNICA
009956 ACAI BERRY E.S. 10% POLIFENOLI

PRODOTTO	ACAI BERRY ESTRATTO SECCO 10% POLIFENOLI
NOME BOTANICO	Euterpe oleracea Mart.
FAMIGLIA BOTANICA	Arecaceae
NUMERO CAS	Euterpe oleracea fruit extract: 879496-95-4 (generic), 906351-38-0 (generic)<br>Maltodextrin: 9050-36-6<br>Aqua: 7732-18-5
PARTE DELLA PIANTA IMPIEGATA	Frutto
SOLVENTI DI ESTRAZIONE	Acqua

ANALISI MATERIA PRIMA	SPECIFICHE
TITOLO	Polifenoli totali >= 10,0% (spettrofotometrico)
RAPPORTO E/D	Fino a titolo dichiarato (ca. 1/10)
ASPETTO	Polvere igroscopica
COLORE	Marrone rosato
ODORE	Caratteristico
SAPORE	Caratteristico
DENSITÀ	Ca. 0,5 g/ml
SOLUBILITÀ	Moderatamente solubile in acqua
PERDITA ALL'ESSICCAMENTO	<= 8,0%
METALLI PESANTI	Totali <= 20 ppm *<br>Pb <= 3 ppm *<br>Cd <= 1 ppm *<br>Hg <= 0,1 ppm *
PESTICIDI	Conforme a Ph. Eur. ed. vigente e/o al Reg. 2005/396/CE e succ. aggiornamenti *
AFLATOSSINE	Aflatossina B1 < 5 ppb; Aflatossine totali (B1, B2, G1, G2) < 10 ppb *
IDROCARBURI POLICICLICI AROMATICI	Conforme (Reg. 1933/2015/CE) *
CARICA BATTERICA TOTALE	<= 5x10000 ufc/g (TAMC, Ph. Eur. 5.1.8, cat. B, uso orale)
LIEVITI E MUFFE	<= 5x100 ufc/g (TYMC, Ph. Eur. 5.1.8, cat. B, uso orale)
PATOGENI	Salmonella: assente/25 g; E. coli: assente/1 g (Ph. Eur. 5.1.8, cat. B, uso orale) *
BATTERI GRAM NEGATIVI RESISTENTI AI SALI BILIARI	<= 100 ufc/g (Ph. Eur. 5.1.8, cat. B, uso orale) *

* Eseguita in base ad un piano di autocontrollo

INFORMAZIONI GENERALI	
NOME INCI	Euterpe oleracea fruit extract<br>Maltodextrin<br>Aqua
NUMERO EINECS/ELINCS	Euterpe oleracea fruit extract: /<br>Maltodextrin: 232-940-4

TECHNICAL DATASHEET
009956 EUTERPE OLERACEA P.E. 10% POLYPHENOLS

PRODUCT	EUTERPE OLERACEA POWDER EXTRACT 10% POLYPHENOLS
BOTANICAL NAME	Euterpe oleracea Mart.
BOTANIC FAMILY	Arecaceae
CAS NUMBER	Euterpe oleracea fruit extract: 879496-95-4 (generic), 906351-38-0 (generic)<br>Maltodextrin: 9050-36-6<br>Aqua: 7732-18-5
PART OF PLANT USED	Fruit
SOLVENT EXTRACTION	Water

RAW MATERIAL ANALISYS	SPECIFICATIONS
ASSAY	Total polyphenols >= 10,0% (spectrophotometric method)
E/D RATIO	Up to stated assay (approx. 1/10)
APPEARANCE	Hygroscopic powder
COLOUR	Pinkish brown
ODOUR	Characteristic
TASTE	Characteristic
DENSITY	Approx. 0,5 g/ml
SOLUBILITY	Sparingly soluble in water
LOSS ON DRYING	<= 8,0%
HEAVY METALS	Total <= 20 ppm *<br>Pb <= 3 ppm *<br>Cd <= 1 ppm *<br>Hg <= 0,1 ppm *
PESTICIDES	Complies with Ph. Eur. current ed. and/or Reg. 2005/396/EC and amendments *
AFLATOXINS	Aflatoxin B1 < 5 ppb; total aflatoxins (B1, B2, G1, G2) < 10 ppb *
POLYCYCLIC AROMATIC HYDROCARBONS	Complies (Reg. 1933/2015/EC) *
TOTAL BACTERIA	<= 5x10000 cfu/g (TAMC, Ph. Eur. 5.1.8, cat. B, oral use)
YEASTS AND MOULDS	<= 5x100 cfu/g (TYMC, Ph. Eur. 5.1.8, cat. B, oral use)
PATHOGENS	Salmonella: absent/25 g; E. coli: absent/1 g (Ph. Eur. 5.1.8, cat. B, oral use) *
BILE- TOLERANT GRAM-NEGATIVE BACTERIA	<= 100 cfu/g (Ph. Eur. 5.1.8, cat. B, oral use) *

* Performed on the basis of the self-control plan

GENERAL NOTICES	
INCI NAME	Euterpe oleracea fruit extract<br>Maltodextrin<br>Aqua
EINECS/ELINCS NUMBER	Euterpe oleracea fruit extract: /<br>Maltodextrin: 232-940-4

FONTANA
standardised natural active principles

SCHEDA TECNICA	TECHNICAL DATASHEET
009956 ACAI BERRY E.S. 10% POLIFENOLI	009956 EUTERPE OLERACEA P.E. 10% POLYPHENOLS

	Aqua: 231-791-2		Aqua: 231-791-2
PROVENIENZA MATERIA PRIMA	Brasile	ORIGIN OF RAW MATERIAL	Brazil
ORIGINE DELLA PIANTA	Pianta spontanea	ORIGIN OF THE PLANT	Spontaneous plant
EPOCA DI RACCOLTA	Da luglio a dicembre	HARVEST TIME	From July to December
ATTIVI DELLA PIANTA	Antocianosidi, fibre, steroli (betasitosteroli), acidi grassi (oleico, omega-9 e linoleico, omega-6), vitamine (B1, B6, C), minerali (ferro e potassio), aminoacidi	ACTIVES OF THE PLANT	Anthocyanins, fiber, sterols (beta-sitosterols), fatty acids (oleic, linoleic and omega-9, omega-6), vitamins (B1, B6, C), minerals (iron and potassium), amino acids
TIPO DI PRODOTTO ED IMPIEGO	Prodotto ad uso professionale con applicazioni in ambito alimentare e cosmetico	TYPE OF PRODUCT AND USE	Product for professional use with food and cosmetic application
COMPOSIZIONE	Euterpe oleracea fruit extract: fino a 100%; Maltodextrin <= 30%; Aqua <= 8%	COMPOSITION	Euterpe oleracea fruit extract: up to 100%; Maltodextrin <= 30%; Aqua <= 8%
ALLERGENI	Esente da allergeni alimentari (Reg. 1169/2011/UE, Alleg. II)	ALLERGENS	Free from food allergens (Reg. 1169/2011/EU, Annex II)
	Non contiene glutine		It does not contain gluten
	Non ci si aspetta la presenza di allergeni cosmetici in quantità significative e non sono aggiunti intenzionalmente		The presence of cosmetic allergens is not expected in significant quantities and they are not intentionally added
GRANULOMETRIA	>= 90,0% passa attraverso 300 micron	PARTICLE SIZE	>= 90,0% through 300 micron
MANIPOLAZIONE	Manipolare in locali provvisti di un buon sistema di aerazione; impedire il contatto delle polveri con sorgenti di accensione, quali fiamme libere, scintille, ecc	HANDLING	Handle in well ventilated room, avoid the contact of powder with fire sources, such as flames and sparks, etc
CONSERVAZIONE	Conservare in contenitori ben chiusi, al riparo da luce, calore e umidità	STORAGE	Store in well closed containers, away from light, heat and moisture
PROPRIETÀ	Antiossidante	PROPERTIES	Antioxidant
	Azione sul metabolismo dei carboidrati e dei lipidi		Action on the metabolism of carbohydrates and lipids
	Regolarità del transito intestinale		It regulates the intestinal transit
	Uso esterno: condizionante cutaneo e per capelli		External use: hair and skin conditioner
BIBLIOGRAFIA	CosIng - European Commission Database	BIBLIOGRAPHY	CosIng - European Commission Database
	Il Nuovo Codex Vegetabilis - G. Proserpio		Il Nuovo Codex Vegetabilis - G. Proserpio
	Dizionario di fitoterapia e piante medicinali - Campanini - Tecniche Nuove		Dizionario di fitoterapia e piante medicinali - Campanini - Tecniche Nuove

ANNOTAZIONI		NOTES	
NOTE	Esente da OGM (Reg. 1829-1830/2003/CE); esente da rischio BSE/TSE	NOTES	GMOs-free (Reg. 1829-1830/2003/EC); BSE/TSE-free
	Non irradiato		Not irradiated
	Non contiene sostanze classificate C.M.R.		It does not contain C.M.R. substances
	Non contiene nanomateriali		It does not contain nanoparticles/nanomaterials
	Non testato sugli animali		It has not been tested on animals
